# Metabolomic Insights of Biosurfactant Activity from *Bacillus niabensis* against Planktonic Cells and Biofilm of *Pseudomonas stutzeri* Involved in Marine Biofouling

**DOI:** 10.3390/ijms24044249

**Published:** 2023-02-20

**Authors:** Ilse Sánchez-Lozano, Luz Clarita Muñoz-Cruz, Claire Hellio, Christine J. Band-Schmidt, Yair Cruz-Narváez, Elvia Becerra-Martínez, Claudia J. Hernández-Guerrero

**Affiliations:** 1 Instituto Politécnico Nacional, Centro Interdisciplinario de Ciencias Marinas, Av. Instituto Politécnico Nacional S/N, Col. Playa Palo de Santa Rita, La Paz 23096, Mexico; 2 CNRS, IRD, Ifremer, LEMAR, Univ. Brest, Institut Universitaire Européen de la Mer, F-29280 Plouzané, France; 3 Laboratorio de Posgrado de Operaciones Unitarias, Instituto Politécnico Nacional-ESIQIE-UPALM, Unidad Profesional Adolfo López Mateos, Edificio 7, 1.er Piso, Sección A, Av. Luis Enrique Erro S/N, Zacatenco, Delegación Gustavo A. Madero, Mexico City 07738, Mexico; 4 Centro de Nanociencias y Micro y Nanotecnologías, Instituto Politécnico Nacional, Unidad Profesional Adolfo López Mateos, Av. Luis Enrique Erro S/N, Zacatenco, Delegación Gustavo A. Madero, Mexico City 07738, Mexico

**Keywords:** *Bacillus*, biosurfactants, ^1^H NMR, metabolomics, *Pseudomonas stutzeri*

## Abstract

In marine environments, biofilm can cause negative impacts, including the biofouling process. In the search for new non-toxic formulations that inhibit biofilm, biosurfactants (BS) produced by the genus *Bacillus* have demonstrated considerable potential. To elucidate the changes that BS from *B. niabensis* promote in growth inhibition and biofilm formation, this research performed a nuclear magnetic resonance (NMR) metabolomic profile analysis to compare the metabolic differences between planktonic cells and biofilms of *Pseudomonas stutzeri*, a pioneer fouling bacteria. The multivariate analysis showed a clear separation between groups with a higher concentration of metabolites in the biofilm than in planktonic cells of *P. stutzeri*. When planktonic and biofilm stages were treated with BS, some differences were found among them. In planktonic cells, the addition of BS had a minor effect on growth inhibition, but at a metabolic level, NADP+, trehalose, acetone, glucose, and betaine were up-regulated in response to osmotic stress. When the biofilm was treated with the BS, a clear inhibition was observed and metabolites such as glucose, acetic acid, histidine, lactic acid, phenylalanine, uracil, and NADP+ were also up-regulated, while trehalose and histamine were down-regulated in response to the antibacterial effect of the BS.

## 1. Introduction

Marine biofilms are composed by the aggregation of many species of bacteria, unicellular algae, and protozoa, which are the initial biological colonizers of new surfaces in the sea [1]. These microorganisms are enfolded in an extracellular polymeric substance (EPS) composed of polysaccharides, proteins, nucleic acids, and lipids [2]. The biofilm pattern of growth in the life cycle of microbes provides specific properties, advantages, and a higher level of organization during colonization than in the free-living (planktonic) bacterial cells [3]. Biofilms confer protection from desiccation, biocides, antibiotics, heavy metals, and ultraviolet radiation [2,4].

In marine environments, biofilms can induce, inhibit, or have no effect on the settlement of larvae and spores of algae [5]. They can also play a key role in the settlement of macroorganisms because most of them need a particular substratum for survival and reproduction [6]. Although it is a natural process, biofilm development can cause negative impacts, including the promotion of the biofouling process [7] and corrosion of marine man-made structures [8], causing significant economic loses [9]. The biofilms of *Pseudomonas* species are frequently found and contribute to an accelerated corrosion of metallic and steel surfaces [10,11]. Their capacity to form biofilm is due to different ranges of adhesion that occur during the initial attachment to a substratum, and flagella and pilli are important to the colonization and microcolony formation of many marine organisms [12].

Therefore, it is important to find new antifouling strategies for inhibiting colonization bacteria, such as *P. stutzeri,* which are capable of adhering to antifouling paints with biocides such as copper [13] or with high resistance to antifouling agents such as tributylin (TBT) [14]. The transition between the planktonic free-living state to biofilm implies many physiological and biochemical changes [15]. Moreover, the responses of planktonic cells and biofilm to stress (antibacterial or antifouling agents) are markedly different. Studies on biofilm models of *P. aeruginosa* showed that a complex regulatory pathway for the coordination and control of the biofilm response is required [16,17,18].

To prevent the biofouling process, antifouling coatings have been developed and are being used [19]. Some studies indicate that the presence of heavy metals such as copper, which has become the most used biocide in antifouling paint, can alter biofilm formation, larval settlement, and retard the biofouling process [20]. Even if the knowledge of antifouling coatings has been developed for many years, the commercial use of a substance that is not toxic to the marine environment is still nonexistent [9]. In this sense, there are some efforts in the search for a substance that can be used for antifouling, obtained from natural marine products with a non-toxic effect [21,22,23,24]. Biosurfactants (BS) are characterized by their ability to reduce the surface and interfacial tensions between individual molecules at the surface and interface, respectively, in both aqueous solutions and hydrocarbon mixtures [4]. Biosurfactants interfere with biofilm formation by changes in cell adhesion, varying the cell surface hydrophobicity, promoting membrane disruption, or inhibiting the electron transport chain [25]. Within microorganisms, the genus *Bacillus* stands out for its ability to produce BS with antibacterial activity [24,26]; various publications have demonstrated the capacity of BS to reduce biofilm formation [27,28].

Biosurfactants have an important role in the different stages of biofilm development. They can inhibit biofilm formation, control planktonic cell growth, and reduce secondary colonization [29]. In our previous works, cell-free supernatants of *Bacillus niabensis* showed promising results against marine biofilm bacteria [24]. To increase the knowledge of bacteria metabolic changes in response to the effect of a marine BS, metabolomic studies can provide data regarding the chemical changes, adaptation features, and responses of bacteria under different stimuli or conditions [15,30]. NMR-based metabolomic studies give comprehensive information that allows researchers to identify and quantify metabolites to determine with better precision the effect of antibacterial agents on planktonic and biofilm metabolism [18,31].

Therefore, the aim of this study was to evaluate the metabolic response of the growth of planktonic cells and biofilm inhibition of *P. stutzeri* by the effect of non-toxic BS obtained from *B. niabensis* using nuclear magnetic resonance spectroscopy (NMR) to identify molecules associated with planktonic cells and biofilm formation and identify the possible action mode of BS as an antifouling agent.

## 2. Results

### 2.1. Isolation and Identification of Marine Forming Biofilm Bacteria

The isolate F37 from sandblast acrylic tile from a marina in Bahía de La Paz, Baja California Sur, Mexico, was identified as *Pseudomonas stutzeri* by 16S ribosomal sequencing and phylogenetic analysis “barcoding”, with a similarity of 98% in BLAST closest matches. The phylogenetic tree alignment by neighbor joining with 1000 bootstrap replicates is shown in the Supplementary Materials (Figure S1).

### 2.2. Biosurfactant Production from Bacillus niabensis

*Bacillus niabensis* was isolated in 2019 and its capacity to produce BS by cell-free culture supernatants was described previously [24]. The crude biosurfactant was obtained from a culture of *B. niabensis*. The qualitative (oil spreading test and drop collapse test) and quantitative emulsion properties (% E.I.) and the yield of the extract with crude biosurfactant are shown in Table 1. As a positive control, sodium dodecyl sulfate (SDS) at 10% was used, and Marine Broth (MB) was used as a negative control. The different tests are based on the droplet destabilization due to the interfacial tension between the hydrophobic surface and the liquid. The results showed adequate biosurfactant activity, which was similar to the positive control.

### 2.3. Effects of Biosurfactant in Growth and Biofilm Inhibition

In the test of antibacterial activity by agar diffusion, the BS was active against *P. stutzeri* at 30 and 50 µg/mL, but not at a higher concentration (100 µg/mL) (Table 2A). *Pseudomonas stutzeri* showed a high capacity for forming biofilm by crystal violet assay and is evident that BS have the capacity to reduce biofilm formation (Table 2B) and the total protein content of the biofilm (Table 2C). The highest total protein content (0.84 mg/mL) was detected in the biofilm matrix of bacteria without BS. The lowest total protein content and growth rate of *P. stutzeri* were observed when BS was added at concentrations of 50 and 100 µg/mL (Table 2D).

**Figure 1:**
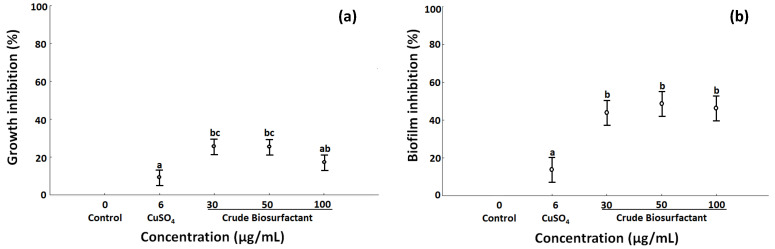
Effects of crude biosurfactant from *Bacillus niabensis* at different concentrations in the growth (**a**) and biofilm (**b**) inhibition of *Pseudomonas stutzeri*, expressed in percentages. Quantification of growth was realized with optical density (OD λ585 nm) in 96-well microplate (*n* = 6 ± SD) and the biofilm by crystal violet assays. Control = bacteria cultured without BS; positive control = CuSO_4_ (6 µg/mL). The same letters indicate no significant differences (one-way ANOVA followed by Tukey’s post hoc test, α ≤ 0.05, *p* = 0.001).

The crude BS from *B. niabensis* at 30 and 50 µg/mL inhibited significantly 30% of the growth of *P. stutzeri* (Figure 1a). In the biofilm inhibition, no significant difference was observed with the concentrations of BS tested, and all concentrations were able to inhibit almost 50% of the biofilm formation (Figure 1b). In both cases, the BS was more efficient than CuSO_4_ at a concentration of 6 µg/mL.

In antibacterial activity, the negative control, i.e., a disk with dissolvent, was not active.

### 2.4. Metabolomic Changes in Planktonic Cells and Biofilm by Effect of Biosurfactant

The planktonic cells (P) and biofilm (B) of *P. stutzeri* were cultured in the absence and presence of crude biosurfactant (P + BS, B + BS) of *B. niabensis* (30 µg/mL) to compare the metabolomic profile after seven days of exposure. In general, the chemical shift of ^1^H NMR spectrum of *P. stutzeri* obtained at 750 MHz between 0.80 and 9.20 ppm presented signals corresponding to 27 metabolites, including 11 amino acids (alanine, aspartic acid, glutamic acid, histidine, isoleucine, leucine, phenylalanine, threonine, tryptophan, tyrosine and valine), 3 sugars (glucose, mannitol and trehalose), 5 organic acids (acetic acid, formic acid, fumaric acid, lactic acid and succinic acid), 3 nucleosides (adenosine, guanosine and uridine), 1 nucleotide (uracil), and 4 other biomolecules (acetone, betaine, histamine and NADP^+^) (Figure S2). The comparison of ^1^H NMR spectrum in the four treatments (B, P, B + BS, P + BS) showed a similar metabolite profile with differences in the intensity and relative abundance of metabolites (Figure S3).

**Figure 2:**
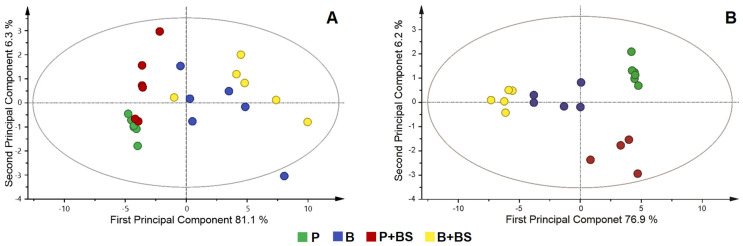
Principal component analysis (PCA) (**A**), orthogonal partial least squares analysis (OPLS-DA) (**B**) score plots of metabolomic profile of planktonic cells (P) and biofilm (B) of *Pseudomonas stutzeri* and with the addition of the crude biosurfactant of *Bacillus niabensis* (P + BS, B + BS).

Metabolic differences between groups were calculated by multivariate analysis. Principal component analysis (PCA) allowed detecting outliers (Figure 2A), while the partial least orthogonal squares discriminant analysis (OPLS-DA) model with projections in two dimensions (PC1 = 76.9% and PC2 = 6.2%) showed an evident separation of the groups (Figure 2B). The validation of the OPLS-DA model is shown in the cross-validation plot (Figure S3). The R^2^ and Q^2^ values in the left were significantly lower than the original points to the right, and Q^2^ regression lines have a negative intersection (R^2^ = 0.0, 0.25; Q^2^= 0.0, −0.70).

**Figure 3:**
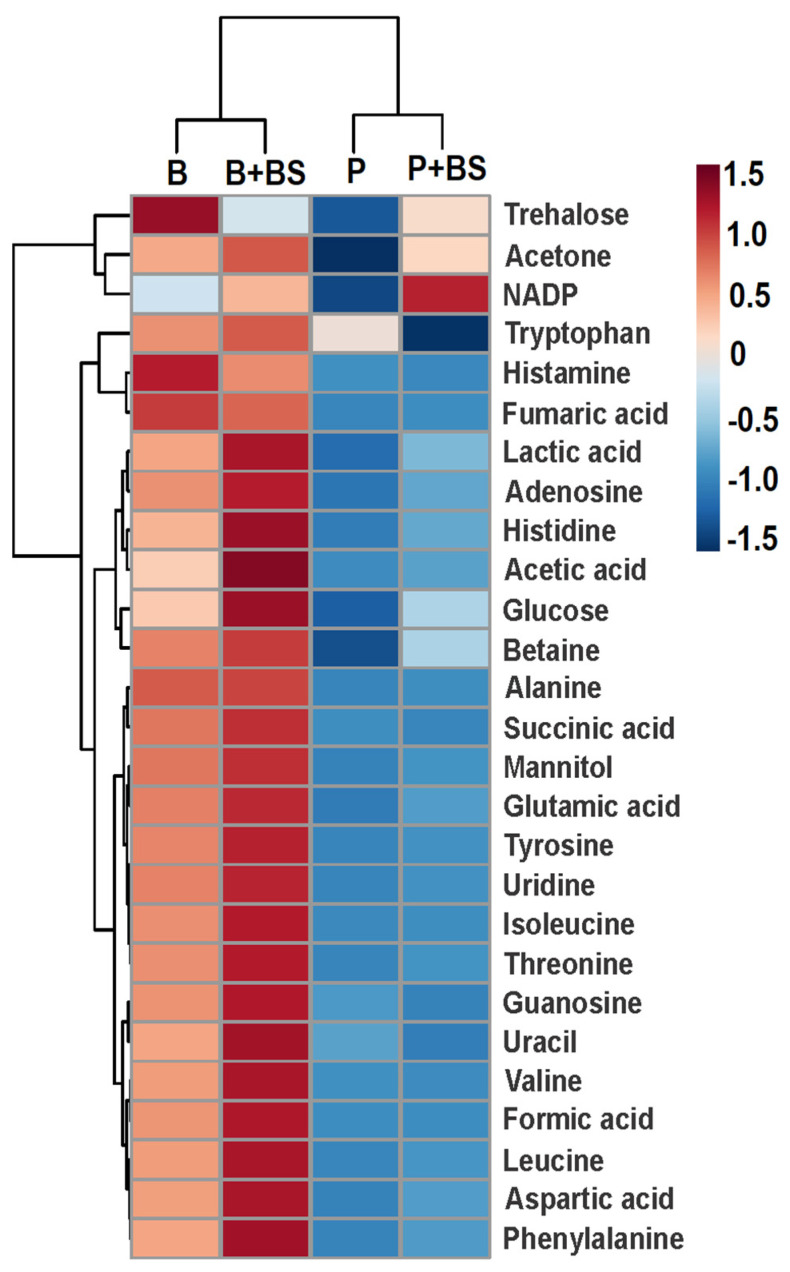
Heat map of 27 metabolites detected in planktonic cells (P) and biofilm (B) of *Pseudomonas stutzeri*. Effect of *Bacillus niabensis* biosurfactant (30 µg/mL) in planktonic cells (P + BS) and biofilm (B + BS). The up-regulated and down-regulated metabolites are shown in red and blue, respectively.

The heat map analysis indicated that the concentration of metabolites varies significantly between the different phases (Figure 3). In planktonic cells, the metabolites are less expressed than in the biofilm. In general, metabolites of planktonic cells are low (below 0.001 Mm) except for tryptophan (0.013 mM) (Figure S4). The difference was more evident in trehalose, histamine, fumaric acid, alanine, and betaine, which had lower concentrations in planktonic cells than in the biofilm. When the planktonic cells were treated with the biosurfactant, the NADP+, trehalose, acetone, glucose, and betaine were up-regulated, and tryptophan was down-regulated (Figure 3).

**Figure 4:**
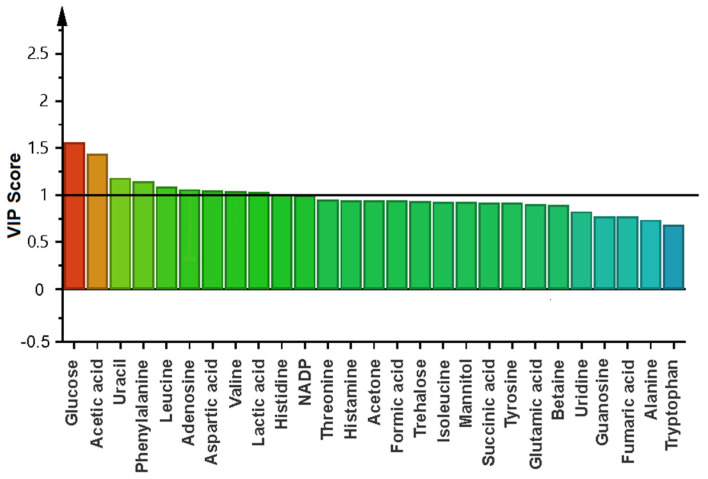
Plot of variable influence on projection (VIP) that identified differential metabolites that contribute to separating the biofilm (B) in the presence and absence of biosurfactant from *Bacillus niabensis*.

To identify the effect of the BS on the biofilm formation, multivariate analysis was performed only with the data of the two groups. Principal component analysis (PCA) in this case did not show outliers, and the partial least orthogonal squares discriminant analysis (OPLS-DA) model with projections in two dimensions (PC1 = 61.2% and PC2 = 12.4%) showed an evident separation of the groups by component 1 (Figure S5). The variable influence on projection statistics (VIP ≥ 1) of OPLS-DA modeling led to the identification of 11 differential metabolites when the bacteria were treated with BS, including carbohydrates, amino acids, organic acids, nucleosides, and other metabolites such as NADP+ (Figure 4). This agrees with the results of the cluster heat map analysis, which indicated that when the biofilm is treated with BS, glucose, acetic acid, histidine, lactic acid, phenylalanine, uracil, and NADP+ were up-regulated, and trehalose and histamine were down-regulated (Figure 3).

## 3. Discussion

As part of biofouling prevention strategies, the use of compounds that inhibit marine pioneer fouling bacteria is being considered—in particular, macrofouling-inducing bacteria such as *Pseudomonas*, which can promote the settlement of organisms such as the marine sponge *Cliona intestinalis* [32]. *Pseudomonas* species have a range of adhesin, which are used in the initial attachment to a substratum, leading to biofilm formation [12,33].

In this work, the pioneer strain isolated from Marina La Paz was identified as *Pseudomonas stutzeri* and showed a high capacity to form biofilm. *Pseudomonas stutzeri* is considered a cosmopolitan species with diverse physiological and biochemical properties [34]. Strains of the genus *Pseudomonas* are characterized by rapid biofilm growth due to the secretion of EPS composed of proteins and polysaccharides, leading to the formation of a mature biofilm in 5–7 days under in vitro conditions [33].

When *P. stutzeri* was exposed to the antifouling compound CuSO_4_ at a usually toxic concentration (6 µg/mL) [24], no significant decrease in growth or biofilm formation was observed compared to the control (bacteria non-treated with CuSO_4_). This may be due to the metabolic properties of *P. stutzeri*, such as denitrification, the ability to fix nitrogen, degradation of pollutants, and interaction with toxic metals [12]. In the search for new non-toxic antifouling compounds, we consider our strain of *P. stutzeri* as a model species to evaluate the antibiofilm and antibiotic properties of crude BS of *B. niabensis.*

The antibacterial activity of *B. niabensis* BS at the minimum concentration tested (30 µg/mL) showed an inhibition diameter of 12.53 mm. Similar results were observed with the BS from *B. circulans* that showed an antimicrobial diameter between 12 and 24 mm against Gram-negative bacteria when tested at 1000 µg/mL [35]. The BS produced by *B. licheniformis* has antimicrobial activity with an inhibition zone of 11–25 mm against Gram-positive bacteria and 10–19 mm against Gram-negative bacteria at 48 µg/mL [36].

When *P. stutzeri* was treated with crude BS of *B. niabensis,* it was possible to reduce growth by 30% and biofilm formation by almost 50%. However, no significant differences were observed at BS concentrations of 30, 50, and 100 µg/mL. There are only a few studies on the antibacterial and antibiofilm activity of *B. niabensis*. The use of *B. niabensis* cell-free supernatants included in experimental marine paint showed acceptable antifouling activity in field assays with low toxicity [24]. Moreover, the development of gold nanoparticles using *B. niabensis* showed strong antibiofilm activity against *P. aeruginosa* with 72% inhibition of biofilm formation, without affecting cell growth [37]. Others BS produced by *Bacillus* genus have antibiofilm activity. For example, BS of *Bacillus* sp. A7 showed 37% inhibition of biofilm at a low concentration (31.25 µg/mL), 46% inhibition when used at 125 µg/mL, and complete inhibition at 500 µg/mL [38].

*Bacillus* species, commonly found in marine environments, produce a large range of low-molecular-weight BS (lipopeptide-type) with broad uses in different biotechnology fields [39,40], including antimicrobial and fungicidal compounds [41]. The crude BS of *B. niabensis* investigated in this research showed an interesting emulsion index with better results than the control SDS. Similar capacities have been highlighted with lipopeptides from *Bacillus licheniformis* [42,43]. The antibiofilm activity of lipopeptides was observed in previous studies; for example, some *Bacillus* strains (*B. subtilis*, *B. amyloliquefaciens*, *B. siamensis*) [44,45,46] produce fengycin, iturin A, and surfactin, which are lipopeptides with antibiofilm activity. This group of compounds had the ability to completely inhibit *S. aureus* biofilm formation at the concentration of 15 mg/mL. Furthermore, they reduced biofilm formation by 50% at concentrations of 1.5 and 0.15 mg/mL [47].

The sufficient yield of bioactive compounds during extraction is an important factor to avoid high costs of production and obtain enough quantity of BS for commercial use. However, our study did not focus on the yield of crude BS production. Despite a low yield in our work (0.15 g/L), using supplemented culture medium is a possibility to optimize the process. In *B. licheniformis,* the yield can increase to 0.86 g/L depending on the carbon sources [48]. In *B. subtilis,* a yield of 4.8 g/L was recorded when using media supplemented with metals (iron, manganese, and magnesium) [49].

BS are a promising antibiofilm and antifouling molecules due to their low toxicity [40], their mode of action (e.g., modulation of the expression of biofilm genes) [47,50] and their amphiphilic nature (that allows them disrupt membrane integrity, leading to cell lysis, linked with the ability to affect adhesion and dislodgement of bacteria from the surface) [51,52]. They can also promote changes in membrane structure, altering the essential membrane structure such as the transport and production of energy [53]. In our work, when the biofilm was treated with *B. niabensis* BS, a reduction in biofilm was recorded as well as a diminution of the total protein, which can be due to the inhibition of protein synthesis [54]. All these interesting properties, as well as the fact that it can be used at a low concentration, make it a promising antifouling alternative [54,55].

The metabolic effect of *B. niabensis* BS towards *P. stutzeri* was studied using a low concentration with activity (30 µg/mL). The signals of the ^1^H NMR spectrum led to the determination of the presence of 27 metabolites from six principal groups in different percentages: amino acids (40.7%), organic acids (18.5%), sugars (11.1%), nucleosides (11.1%), nucleotides (3.7%), and others (14.8%). Although the presence of all these metabolites was equal in both phases, the quantification was different. The results of the multivariate analysis OPLS-DA showed a clear separation between groups of planktonic cells and biofilm state. The validation plots (Figure S5) show that the R_2_ and Q_2_ values on the left are significantly lower than the original points on the right, and Q_2_ regression lines have a negative intersection (R^2^=0.0, 0.5; Q^2^=0.0, −0.7). These values indicate that the OPLS-DA model is robust and not random and without over-fitting [56].

It has been stated that planktonic and biofilm phases have different metabolic activity profiles resulting from the cell attachment onto surfaces [57]. It is important to consider this difference when developing new antibacterial agents [58]. The metabolites have higher concentrations in the biofilm of *P. stutzeri* than in planktonic cells, indicating higher metabolic activity. Trehalose, alanine, histamine, and fumaric acid are more expressed in the biofilm. These metabolites and other carbohydrates may play a role in composing the biofilm matrix as components of the extracellular matrix. The presence of high sugar levels in the biofilm may also be related to the constitution of the EPS matrix. Trehalose was found in significantly higher concentrations in biofilms compared to suspended cultures. In *P. aeruginosa*, high levels of these carbohydrate-related metabolites have the potential to uncover the presence of biofilm under multiple growth conditions [17]. Alanine metabolism has been reported to be crucial for adhesion and biofilm formation, because of its role in the formation of cell wall peptidoglycan [59]. Organic acids are bound to the metabolites of biofilms, especially mature biofilms [60].

When the BS was utilized to inhibit both planktonic cells and biofilm, different effects were recorded. The addition of BS to planktonic cells resulted in the up-regulation of NADP+, trehalose, acetone, glucose, and betaine, and the down-regulation of tryptophan. Trehalose is a biocompatible compound that has the function of osmolytes and helps an organism to survive under osmotic stress. In studies with *P. aeruginosa*, trehalose has the function of promoting the acquisition of nutrients that allow the replication of the bacteria [61]; like trehalose, betaine is used as an energy resource under osmotic stress [62]. Similar results were observed in *Mycobacterium tuberculosis*—when persister bacilli were treated with an antibiotic, the trehalose metabolism and glycolysis were altered in a metabolic effort to acquire drug tolerance [63].

When the biofilm was treated with the BS, metabolites such as glucose, acetic acid, histidine, lactic acid, phenylalanine, uracil, and NADP+ were up-regulated while trehalose and histamine were down-regulated. The bacteria metabolic changes depend on the antibacterial compound characteristics. When biofilms of *P. aeruginosa* were treated with combinations of ciprofloxacin with baicalein and esculin hydrate, the uracil concentration was increased, and this was related to pyrimidine synthesis [64], which has an important role in many functions of *P. aeruginosa* [65] in response to antibacterial compounds. With respect to compounds that were down-regulated, contrary to the behavior observed in the planktonic phase, in the biofilm, the BS caused a decrease in the concentration of trehalose. This could be because during the formation of biofilm of some bacteria, trehalose is down-regulated due to its relationship with the central carbon metabolism [66]. Moreover, the decrease in trehalose may be related to its part in the formation of NADP, which plays an important role as a reducing agent before the effect of an antibacterial compound [63].

The results of this research showed that *B. niabensis* biosurfactants reduce growth and biofilm formation of *P. stutzeri* with a concomitant metabolic change in response to antibacterial activity. This provides new information regarding the inhibitory mechanism of the BS against pioneer marine fouling bacteria.

## 4. Materials and Methods

### 4.1. Chemical and Strain Identification

The chemicals used for the identification of marine bacteria were of molecular biology grade. For culture and assays, they were of analytical grade, obtained commercially. For ^1^H NMR, deuterated dissolvents were used.

*Bacillus niabensis* was isolated from the marine sponge *Mycale ramulosa* and was selected due to its high biosurfactant activity in the cell-free supernatant. The identification was performed by partial sequencing of the 16S rRNA region (GenBank accession number MT887632) [24].

The bacteria involved in the marine biofilm, *P. stutzeri,* was collected and isolated from the Marina La Paz, Bahía de La Paz, Mexico (24°08′32″ N–110°18′39″ W). A sandblasted acrylic tile (6 × 12 cm) was immersed at 1 m depth (November 2020). After 48 h, the tile was placed in a plastic bag and transported to the laboratory, where it was washed with seawater in sterile conditions. The tile was rubbed with a swab and placed in 10 mL of saline solution (SSS) (NaCl 2.5%). With the bacterial suspension, a serial dilution was prepared (10^−1^ to 10^−5^). Additionally, 100 µL of the bacterial suspension was spread on plates of marine agar in triplicate. Bacterial colonies were isolated and characterized based on morphology and Gram staining.

The biofilm formation of the F37 strain was evaluated by a crystal violet assay [67] with some modifications. To a 96-well flat bottom polystyrene microtiter plate, 10 µL of cell suspension (OD_585_ 1) was added, previously inoculated with 190 µL of Marine Broth in each well per triplicate. In peripheral wells, 200 µL of SSS was added. Cells were incubated 48 h at 35 °C, planktonic cells were removed, and the biofilm was fixed with 99% methanol. The plates were washed twice with SSS and air dried. The crystal violet (0.2%) was added, 200 µL per well; after 5 min, the dye was removed, and the plate was washed twice and air dried. The crystal violet was dissolved in acetic acid (33%), and finally, the biofilm growth was monitored with a microplate reader (OD_585_).

The strain F37 was identified by partial sequencing of 16S rRNA. From 24 h massive culture, genomic DNA was extracted and then purified. PCR was performed using specific oligonucleotides for the 16s rDNA gene [68]. The PCR reaction was run with positive and negative controls (genomic DNA mixture bacterial and oligonucleotides + sterile water without DNA, sterile water + DNA without oligonucleotides). In an agarose gel (2%), the amplification products were separated by electrophoresis and visualized on a UV transilluminator. The bands with the specific amplification were cut and ADN was purified with Zymo Clean Gel Recovery kit (Catalogue No. D4021/D4022, CA, USA). The ADN sequencing was carried out in the IBT Sanger service (UNAM). The sequences were analyzed in BLASTN, and to obtain the phylogenetic analysis, the alignment of the obtained sequences with the reference sequences were determined by BLASTN with MUSCLE. The alignments were analyzed by two methods: neighbor joining and Tamura–Nei parameters. The statistical support was 1000 bootstrap replicates.

### 4.2. Culture and Biosurfactant Properties of Bacillus niabensis

#### 4.2.1. Inoculum and Culture Conditions

After selecting the biosurfactant producer bacteria, *B. niabensis* was cultivated for 24 h (37.5 °C) in TSA medium (2.5% NaCl). A cell suspension in SSS (DO_585_ = 1) was used to inoculate (2:100 *v*/*v*) 250 mL Erlenmeyer flasks containing 50 mL TSB (2.5% NaCl) medium per triplicate. After seven days of culture in TSB medium (35 °C, 160 rpm), the medium was centrifuged (3000 rpm, 30 min, 4 °C) to obtain the cell-free culture supernatant [69]. Thus, the biosurfactant activity was determined by three methods: (1) oil displacement test, (2) drop collapse test, (3) emulsification assay.

#### 4.2.2. Drop Collapse Test

In a 96-well microtiter plate lid, 2 µL of mineral oil was spread out to the well region delimited as Youseef et al. [70] describes. After one hour at room temperature, 5 µL of supernatant was applied over the oil-coated regions per triplicate. The drop size was observed after 1 min, and the results were considered positive when the drop diameter was at least 1 mm larger that the negative control (distilled water). As a positive control, SDS at 10% was used.

#### 4.2.3. Oil Displacement Test

In a plastic Petri dish with 20 mL of distilled water, 20 µL of mineral oil was added, followed by 10 µL of the supernatant added to the oil surface. Immediately, the diameter of the oil-free clearance zone was measured. The results were positive when the oil was displaced. Distilled water was used as a negative control and SDS at 10% was used as a positive control [71].

#### 4.2.4. Emulsification Assay

The potential of the biosurfactant to emulsify toluene was carried out via an emulsification test [72]. In a tube, 2 mL cell-free supernatant and 2 mL toluene were mixed by vortex for 2 min and left to stand for 24 h. After 24 h, the calculation of the emulsification index (E.I.) was determined by the following equation:

$$\%\mathrm{IE}=\frac{\mathrm{Height}\;\mathrm{of}\;\mathrm{formed}\;\mathrm{emulsion}}{\mathrm{Total}\;\mathrm{height}\;\mathrm{of}\;\mathrm{the}\;\mathrm{solution}}\times 100$$

Distilled water was used as a negative control and SDS at 10% was used as a positive control. The emulsion stability was determined by the volume of the emulsion layer at 0, 24, and 48 h. This test was carried out in triplicate.

### 4.3. Assay of Effect of BS in the Growth and Biofilm Formation

#### 4.3.1. Production and Extraction of Crude Biosurfactant

The crude biosurfactant was isolated according to Ghibri and Ellouze-Chaabouni [73] with some modifications. A flask with 100 mL TSB medium was inoculated (2:100) with an overnight culture of *B. niabensis* (DO = 1). After 48 h of culture at 35 °C, 160 rpm, the supernatant was precipitated overnight at 4° C with HCl 5 M to pH 2. After 24 h, the pellet was collected by centrifugation (3500 rpm at 4 °C for 30 min). The pellet was washed twice with acid water (pH 2), centrifuged (3500 rpm at 4 °C for 30 min), and re-suspended in 25 mL deionized water. The pH was adjusted to 7 with NaOH (5 M). The crude BS was stored at −80 °C, lyophilized, and the yield was registered.

#### 4.3.2. Antibacterial and Antibiofilm Activity

##### Antibacterial Assay by Agar Well Diffusion Method

The biosurfactants dissolved in a mixture of CHCl_3_:MeOH (1:1), to obtain different concentrations (30, 50, and 100 µg/mL), were applied to 6 mm diameter paper disks. An overnight culture of *P. stutzeri* was inoculated on marine agar plates. After, the disks with BS were placed in the plates. Disks impregnated only with dissolvent were utilized as a negative control. The zone of inhibition was measured after incubating at 35 °C for 24 h. All tests were performed in triplicate and the diameter of the inhibition zone represented the mean value (mm) ± SD.

##### Biofilm Formation

The overnight culture of *P. stutzeri* was inoculated in 20 mL marine medium and transferred to the Petri dish with a sterile slide. After 48 h of incubation at 35° C, the slide was gently washed with sterile distilled water and dyed with crystal violet (0.2%) for 10 min. The slides were washed with distilled water and observed under a microscope. 

##### Quantitation of Biofilm Protein

The overnight culture of *P. stutzeri* was inoculated in 20 mL of marine medium and transferred to the Petri dish per triplicate. The treatments included BS at different concentrations (30, 50, and 100 µg/mL). After incubation at 35 °C for 48 h, planktonic cells were removed, and the Petri dish was gently washed with a sterile saline solution (2.5%). The biofilm cells were collected and centrifuged. The supernatant was discarded, and biofilm pellets were resuspended in sterile water, lysed by sonication, and subsequently centrifuged. Total proteins were quantified by Bradford assay [74], and bovine serum albumin was used as a standard.

##### Effect of BS in Growth Rates of *P. stutzeri*

The growth rate assay was performed in a microplate. *Pseudomonas stutzeri* were grown in marine medium, and the treatments contained BS at different concentrations (30, 50, and 100 µg/mL). The microplate was incubated (35 °C) in static conditions, and the optical density at 620 nm was measured every hour. The data were recorded, and the growth rate was calculated as described by Widdel [75].

##### Growth and Biofilm Inhibition

Crude BS of *B. niabensis* was used to evaluate the growth and biofilm inhibition of the biofilm-forming bacteria *P. stutzeri*. The strain was cultivated on marine agar at 35 °C for 24 h and further adjusted to DO = 1 in SSS (2.5%) to yield a bacterial suspension.

The BS was diluted in Marine Broth and aliquoted to have three concentrations (30, 50, and 100 µg/mL). In a 96-well flat bottom microplate (Costar 3596, Corning, Corning, NY, USA) with six replicates, the BS (20 µL) at different concentrations was added in each well and finally inoculated with 180 µL of bacterial suspension. As a positive control, the bacteria were cultured only in the Marine Broth, and as a negative control, 6 µg/mL copper sulfate (CuSO_4_) was used. The plates were incubated for 48 h at 35 °C. After recording the optical density at 620 nm in a microplate reader, the growth of the bacteria in the presence of BS was evaluated. To evaluate the biofilm formation, the crystal violet protocol described by Shukla and Rao [67] was performed. The results were expressed as percentages.

### 4.4. ^1^H NMR Metabolic Analysis

#### 4.4.1. Biofilm and Planktonic Cells Source and Sample Preparation

The biofilm and planktonic cells were obtained in accordance with Mikkelsen et al. [76], with some modifications. *Pseudomonas stutzeri* was cultured on marine agar plates for 24 h. Then, the cells were adjusted to DO = 1 (saline solution = 2.5%, 585 nm) and inoculated in a Petri dish with Marine Broth (1:9). In seven replicates, 30 µg/mL of BS was added. Seven replicates were incubated without BS (control) for seven days at 35 °C. After the incubation period, the planktonic cells were removed with a micropipette and recovered by centrifugation (12,000 rpm, 4 °C, 20 min). Biofilm cells were removed from the Petri dish with a cotton swab and PBS solution, and recovered by centrifugation (12,000 rpm, 4 °C, 20 min). Samples were stored at −80 °C and lyophilized. All samples were extracted with a mixture of KH_2_PO_4_ buffer in deuterium oxide (D_2_O). The mixture was mixed in a vortex, and the extraction was realized in an ultrasonic bath for 30 min and then centrifuged for 20 min at 4200 rpm. The supernatants were transferred into NMR tubes (5 mm).

#### 4.4.2. Nuclear Magnetic Resonance (NMR) Experiments and Data Analysis

The NMR experiments were performed in a Bruker 750 MHz spectrometer (Bruker Biospin, Rheinstetten, Germany) equipped with a 5 mm TXI cryoprobe. The aqueous extracts from the biofilm and planktonic cells were measured at 298.1 ± 0.1 K without rotation, and with four dummy scans prior to 64 scans. Acquisition parameter were set as follows: FID size = 64 K, spectral width = 19.9967 ppm, receiver gain = 1, acquisition time = 2.18 s, relaxation delay = 10 s, mixing time = 100 ms, FID resolution = 0.45 Hz. Data acquisition was achieved by using a NOESY pre-saturation pulse sequence (Bruker 1D noesypr1d) with water suppression via selective irradiation of the water frequency during recycling and mixing time delays [77].

The NMR data were processed in accord with Liu et al. [78]. Fourier transform and baseline correction were realized on all data. The TSP shift signal was adjusted to 0.00 ppm using TOPSPIN 2.1 software (Bruker Biospin GmbH, Rheinstetten, Germany). The residual signal of water (δ 4.75–4.90 ppm) was suppressed by using MestReNova software (version 6.1.0, Mestrelabs Research SL, Santiago de Compostela, Spain). Finally, these data were converted into SIMCA-P version 14.0 (Umetrics, Umea, Sweden).

#### 4.4.3. Identification and Quantification of Metabolites

For the identification of metabolites, the software Chenomx was utilized and the chemical shift and coupling constant of the signals contrasted with the NMR spectra available in the Biological Magnetic Resonance Data Bank (BMRB; www.bmrb.wisc.edu) and the Human Metabolome Data Base (HMDB; http://www.hmdb.ca/). The quantification of compounds was realized by integration of the ^1^H NMR signals, using TSP as the internal standard [79]. The intensity of a signal in the ^1^H NMR spectrum is proportional to the molar concentration of metabolites [78,80,81].

#### 4.4.4. Statistical Analysis

For concentrations of metabolites, the mean and standard deviation were calculated. A one-way ANOVA was carried out using Statistica software to determine significant differences in metabolite levels. Multiple-comparison tests were performed to reveal pair-wise differences between means (*p* < 0.05). *p* value *<* 0.05 was considered statistically significant.

The multivariate analysis was realized in SIMCA-P version 14.0 (Umetrics, Umea, Sweden). Principal component analysis (PCA) was applied to analyze intrinsic variation in the dataset. All the variables were Paretto-scaled for multivariate analysis. The variables were subjected to orthogonal partial least squares discriminant analysis (OPLS-DA) to identify differential components among samples. A hoteling T2 region showing an ellipse in score plots of the model, was used to define the 95% confidence interval [82]. Validation of the model was realized using permutation tests (200 times). The quality of the model was determined by R_2_ and Q_2_ values [83]. A cluster heat map was created to visualize the abundance of metabolites in planktonic cells and biofilm of *P. stutzeri* with and without BS.

## 5. Conclusions

The BS from *B. niabesis* has the capacity to reduce biofilm formation and cellular growth in the planktonic stage of *P. stutzeri*. This interaction promotes metabolomic changes in response to osmotic stress and protects the cells against the antibacterial activity of BS. We conclude that the crude BS of *B. niabensis* can act as a disruptor to the exopolysaccharide matrix in biofilm (the OD in the microplates with BS was lower than in the plates with bacteria without BS) and cause metabolic stress to cells in the biofilm and planktonic stages. As biofilm formation and metabolomic composition are key in the aggregation of macrofoulers, this BS could be a suitable candidate for inhibiting the biofilm formation of pioneer marine bacteria.

## Figures and Tables

**Table 1:** Yield and biosurfactant potential of *Bacillus niabensis* in oil spreading and drop collapse tests, emulsification properties, and yield.

Bacteria	Gram Reaction	Oil Spreading Test (mm)	Drop Collapse Test (mm)	Emulsification Index (% EI24) (Toluene)	Yield (mg/L)
*Bacillus niabensis*	Bacillus Gram+	Positive 5.00 cm	Positive 9.34 mm	69.33 ± 1.44	147.00
Controls					
MB	-	0	3.60	10.70 ± 1.20	-
SDS (10%)	-	5.00 cm	11.76	59.00 ± 0.90	-

MB = Marine Broth (negative control), SDS = Sodium Dodecyl Sulfate (positive control).

**Table 2:** Effect of crude biosurfactant from *Bacillus niabensis* at different concentrations in the growth inhibition (A), biofilm formation (B), total protein content (C), and growth rate (D) of *Pseudomonas stutzeri* (*n* = 3 ± SD).

	(A)	(B)	(C)	(D)
Biosurfactant Concentration (µg/mL)	Antibacterial Activity Zone Diameter (mm)	Biofilm Formation	Biofilm Total Protein (mg/mL)	Growth Rates (µ)
0		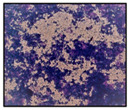	0.84 ± 0.04	0.044
30	12.53 ± 1.40 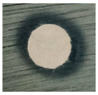	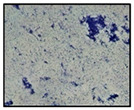	0.68 ± 0.07	0.041
50	9.80 ± 1.91 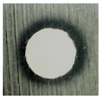	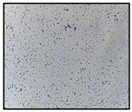	0.47 ± 0.18	0.033
100	0 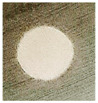	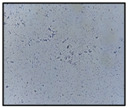	0.35 ± 0.08	0.027

## Data Availability

The data presented in this study are available on request from the corresponding author.

## Supplementary Materials

The following supporting information can be downloaded at: https://www.mdpi.com/article/10.3390/ijms24044249/s1.
