# Supplementary material for: Metabolomic Insights of Biosurfactant Activity from Bacillus niabensis against Planktonic Cells and Biofilm of Pseudomonas stutzeri Involved in Marine Biofouling
Source: Int J Mol Sci. 2023 Feb 20;24(4):4249. doi: 10.3390/ijms24044249 (PMC9965525; doi:10.3390/ijms24044249)
Supplement: Supplementary file 1 [file ijms-24-04249-s001.zip › ijms-2147648-supplementary.pdf]

Suplementary material

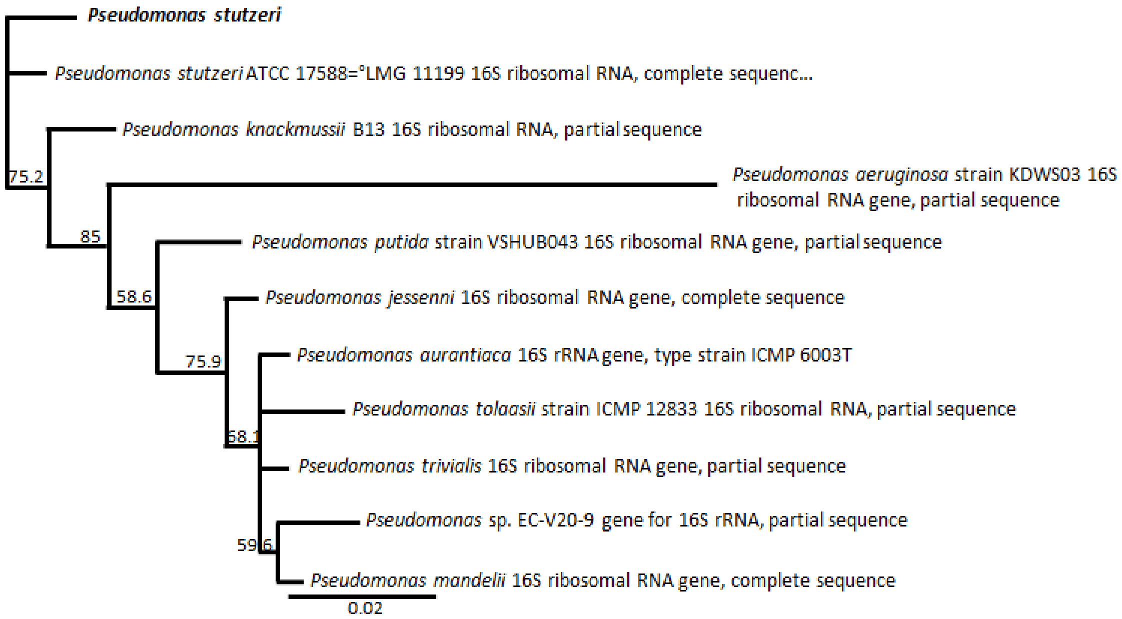

Figure S1. Phylogenetic tree analysis of *Pseudomonas stutzeri*. Based on the 16S rNA gene using Neighbor-Joining (NJ) and Tamura-Nei distance model. The number above branches indicate bootstrap support values.

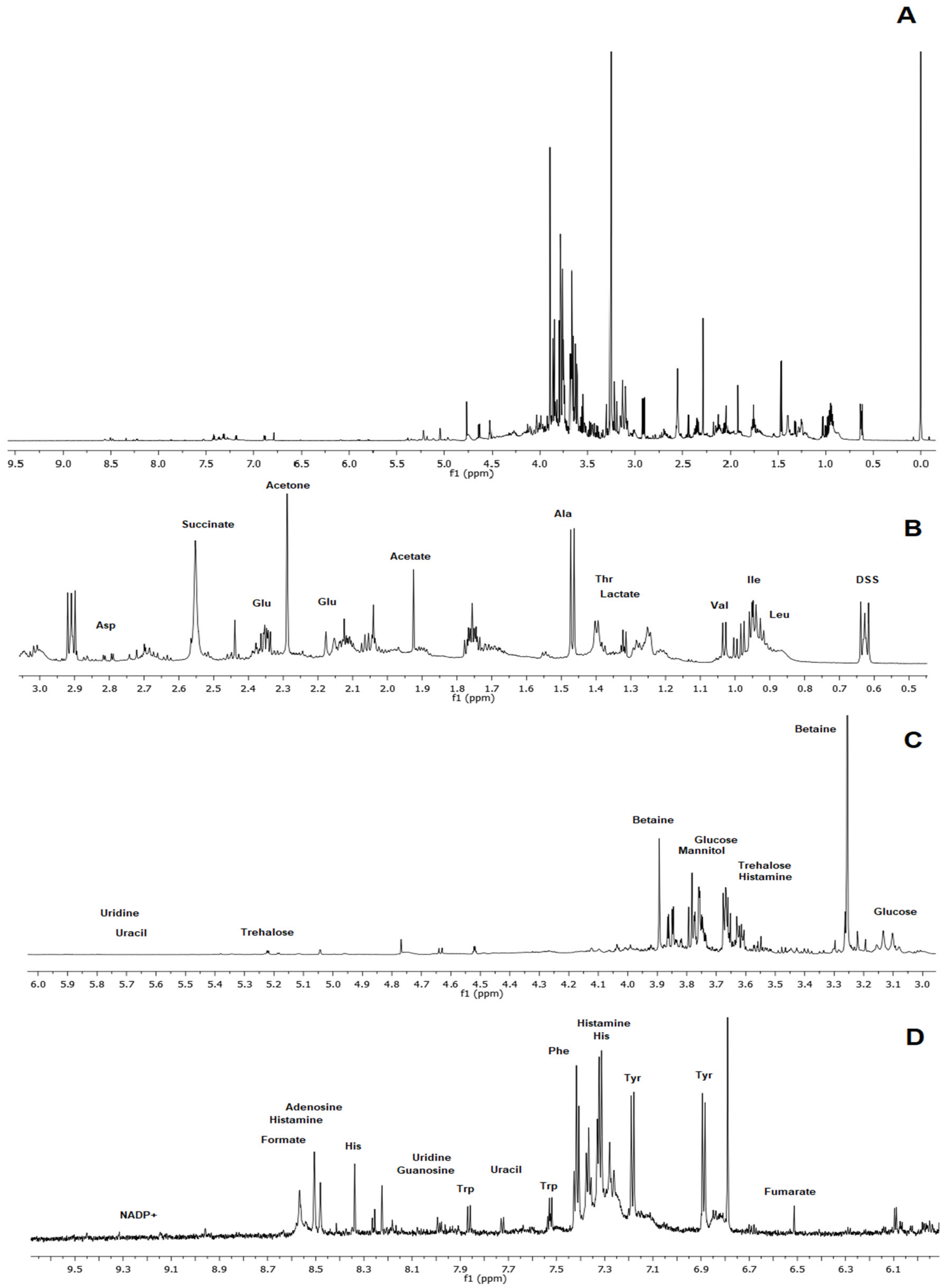

Figure S2. ^{1}H-NMR spectrum obtained at 750 MHz from aqueous extracts of *Pseudomonas stutzeri*.

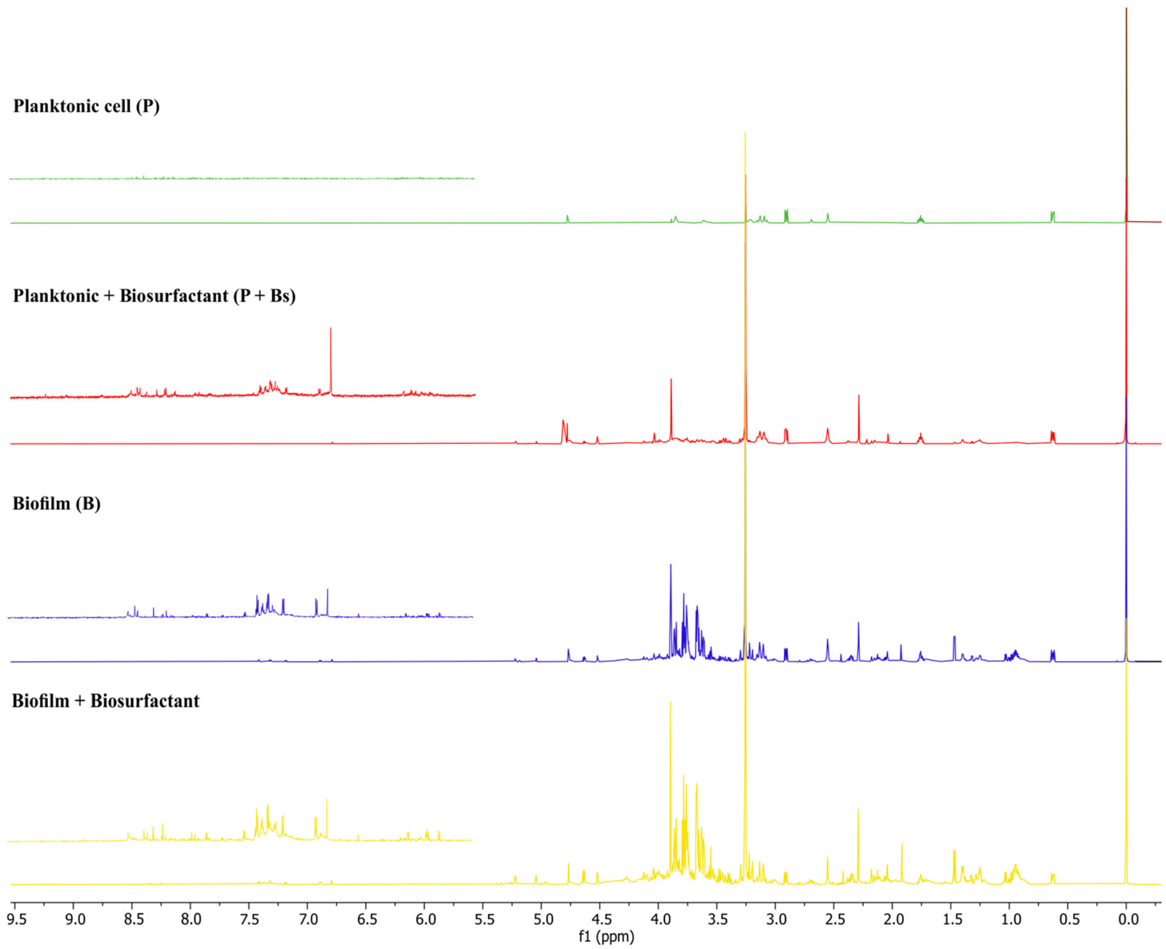

Figure S3. ^{1}H-NMR spectrum of planktonic cells (P), biofilm (B) of *Pseudomonas stutzeri* and with the addition of crude biosurfactant of *B. niabensis* in both conditions (P+BS, B+BS).

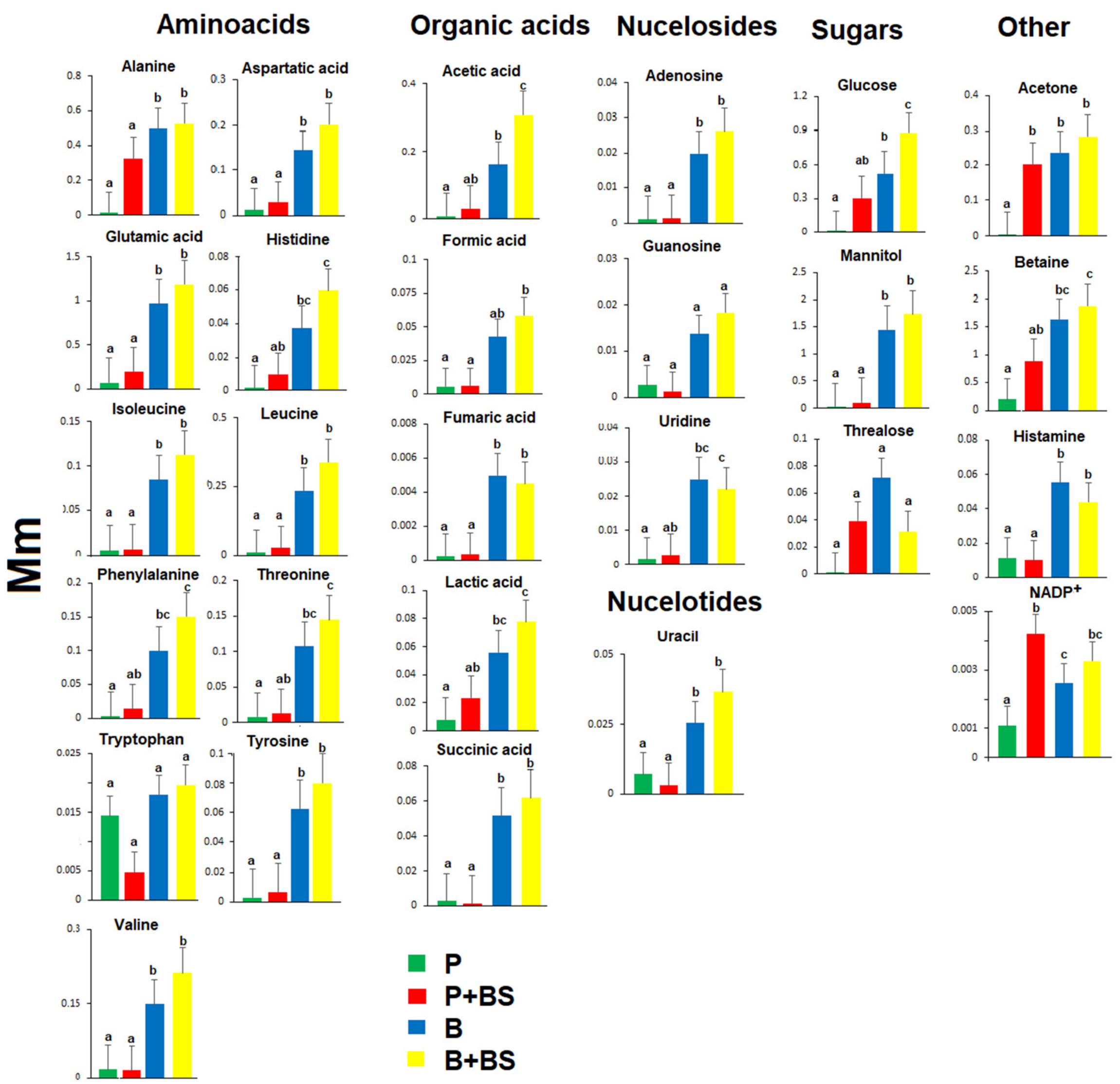

Figure S4. Relative concentration of metabolites presents in plaktonic cells (P), biofilm (B) of *Pseudomonas stutzeri* and with the addition of crude biosurfactant of *B. niabensis* in both conditions (P+BS, B+BS).

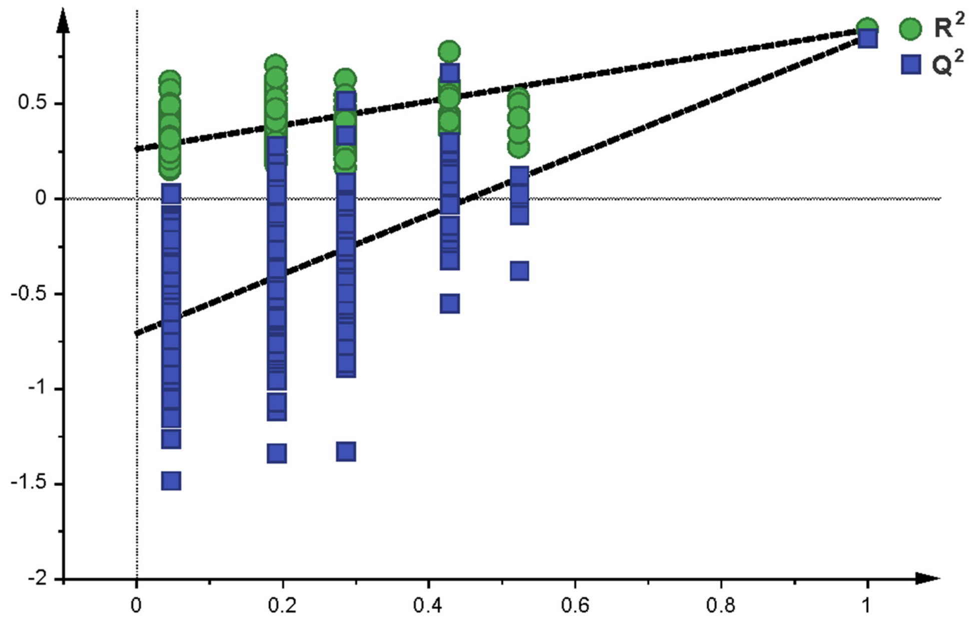

Figure S5. Cross-validation plot of the OPLS-DA model in the different conditions of culture of *Pseudomonas stutzeri*. Plaktonic cells (P), biofilm (B) and with the addition of crude biosurfactant of *B. niabensis* in both conditions (P+BS, B+BS). (Values intercepts, R^2= 0.0, 0.25; Q^2= 0.0, −0.70).
